# Improving knowledge translation in Uganda: more needs to be done

**DOI:** 10.11694/pamj.supp.2014.17.1.3482

**Published:** 2014-01-18

**Authors:** Juliet Nabyonga Orem, David Kaawa Mafigiri, Harriet Nabudere, Bart Criel

**Affiliations:** 1 WHO Uganda office, Health Systems and Services Cluster P. O. Box 24578, Kampala, Uganda; 2 Makerere University School of Social Sciences; P.O Box 7072, Kampala, Uganda; 3 Regional East African Community Health (REACH) Policy Initiative, Uganda and Support the Use of Research Evidence (SURE) for Policy in African Health Systems Project; College of Health Sciences, Makerere University Medical School, P.O. Box 7072 Kampala, Uganda; 4 Institute of Tropical Medicine Antwerp-Belgium; Nationalestraat 155; 2000 Antwerp; Belgium

**Keywords:** Knowledge translation, public health policy, low income countries, Uganda

## Abstract

**Introduction:**

Meeting the health-related Millennium Development Goals in Africa calls for better access to and higher utilisation of quality evidence. The mechanisms through which research evidence can effectively guide public health policy and implementation of health programmes are not fully understood. Challenges to the use of evidence to inform policy and practice include the lack of a common understanding of what constitutes evidence and limited insight on the effectiveness of different research uptake activities. Available Knowledge Translation (KT) models have mainly been developed in high income countries and may not be directly applicable in resource-limited settings. In this study we examine the uptake of evidence in public health policy making in Uganda.

**Methods:**

We conducted a cross-sectional qualitative study consisting of in-depth interviews with 17 purposively-selected health policy makers and researchers. The study explored respondents’ perceptions of the role of evidence in public health policy development, their understanding of KT and their views on the appropriateness of different KT activities that are currently implemented in Uganda.

**Results:**

Although all respondents stated that evidence should inform health policies and programmes, they noted that this occurred infrequently. We noted a lack of conceptual clarity about KT and what precisely constitutes evidence. Respondents reported having been involved in different KT activities, including partnerships and platforms created for knowledge sharing between researchers and end users, but with very mixed results.

**Conclusion:**

There is need for conceptual clarity on the notion of KT and an understanding of the most appropriate KT strategies in low-income settings.

## Introduction

Most African countries are unlikely to meet the health-related Millennium Development Goals’ (MDGs) targets by 2015 [1, 2]. Accelerated progress can only be realised if the coverage of effective health interventions is scaled up. However, this remains a challenge partly as a result of weak health systems [3]. Existing evidence on validated interventions to strengthen the health system rarely informs health policy development and programming. Uptake of evidence in public health policy development and programme implementation has been a subject of research, mainly in high income countries. Several facilitating factors for knowledge translation (KT) have been documented including timely availability of good quality evidence, credibility of researchers, effective interactions between researchers and policy makers’, availability of funding to implement research recommendations, and effective dissemination of evidence, among others [4, 5].

Efforts to improve research uptake have involved the development of models that can explain interactions between stakeholders and the evidence generated, and relationships between evidence and policy processes. Armstrong and colleagues [6] defined several models among which is the linear model which posits that evidence will lead to action. They argued that evidence that responds to identified knowledge gaps will guide policy [6]. The linearity model, however, does not take into consideration other factors that influence policy development, such as the political context and external influence. Enlightenment models highlight the importance of gradual sedimentation of ideas which over time may lead to change [7, 8]. Enlightenment models assume that policymakers stay in office for a fairly long time to allow for sedimentation; however, frequent turn-over of staff in policy making positions limits this model. Political and tactical models, where research is used by policy makers to justify government positions or to reduce the pressure to respond to a given problem, have been criticised for putting emphasis on research processes as opposed to getting evidence into policy [9]. Other KT models have focused on linkages between stakeholders, dissemination modalities of evidence and structures for decision making without adequate attention to the peculiar context of LIC [10, 11]. These peculiarities as pointed out by Young [12] include the chaotic policy making process characterised by donor influence, exaggerated role of civil society, and limited supply of good quality research.

Among the frameworks specifically proposed for low-income countries (LIC) is the Council on Health Research for Development (COHRED) framework encompassing five components namely; 1) research generation and decision making; 2) stakeholders involvement; 3) the mediators who help to link research and policy processes; 4) the research products – consideration for a series of different outputs beyond the research report; and 5) the larger context within which the decision-making and research processes take place [13]. This framework, however, does not specify capacity and institutional requirements for the framework to be applied. The Regional East African Community Health (REACH) policy initiative was established as a knowledge broker to bridge the gap between research and health policy decision making in East African countries. Efforts under REACH focused more on brokering between researchers and policy makers [13, 14]. However, we note that uptake of evidence takes more than dissemination and linkage. Several KT activities have been tried in LIC including putting in place platforms bringing together researchers and policy makers, dissemination in various forms, use of policy entrepreneurs, building capacity of implementers to implement research recommendation but with varying results [5, 13, 15, 16]. Several terminologies have been used to describe the research to action process among which is research application, getting evidence into policy and practice, evidence application, and ‘making that leap between what we know and what we do’. The lack of appreciation of the role of evidence [4] and the lack of common terminology to describe the research to action processes have been cited in literature as possible hindrances to KT [17]. In this paper, we adopt the Canadian Institute for Health Research's (CIHR) definition of KT as “a dynamic and iterative process that includes synthesis, dissemination, exchange and ethically sound application of knowledge to improve health, provide more effective health services and products and strengthen the health care system”[18] This implies that KT is a process spanning the pre-evidence generation stage, evidence generation, synthesis, dissemination to application. Although this definition is widely accepted, there is a lack of clarity of the conceptualization of KT and multiple definitions still exists [17, 19]. Lack of conceptual clarity not only poses a challenge to putting in place mechanisms and activities to promote KT, but also monitoring the extent to which evidence is taken up into policy. Tetroe and colleagues underscored the need to understand the effectiveness of different KT activities [20].

Improving uptake of evidence in public health policy development and programming in low-income settings requires the generation of context-specific evidence on effective KT activities. Understanding stakeholders’ perspectives of the KT process is a starting point. This study investigates the importance of evidence in public health policy making and programming in Uganda, and assesses stakeholders’ conceptualization of KT and involvement in different KT activities.

## Methods

### Study design

We conducted a cross-sectional qualitative study comprising in-depth interviews with key informants (KI) to explore their perceptions on the importance of evidence in public health policy development and programming, their understanding of KT, and their involvement in different KT activities.

### Participants

Respondents included 15 health policy makers and two researchers who were purposively-selected on the basis of their day-to-day involvement in policymaking or research in health systems. All policy makers were members of the Health Policy Advisory Committee (HPAC), the policy advisory body for the health sector. The HPAC comprises of senior government officials from the central and district levels, and representatives of donor agencies, civil society organisation (CSOs), private not for profit (PNFP) organizations and the private-for profit sector (PFP). Details of selected respondents are shown in Table 1.

**Table 1:** Key informant respondents

Sector		No. in HPAC	No. selected
Public	Ministry of Health (5)		
	Central level	9	4
	District level	1	1
	Researcher from School of Public Health*	-	1
Private	Private not for profit (Civil society) (4)		
	Facility based	2	2
	Non facility based	2	2
	Researcher*	-	1
	Private for profit	1	1
Donors	Bilateral	4	2
	Multilateral	3	3
Total			17

HPAC: Health Policy Advisory Committee

* Researchers are not members of HPAC

### Procedures

Interviews followed a guide that included probes on the informants’ perception of the role of evidence in health policy development and programme design, their conceptualization of KT, and their involvement in various KT activities. The interview guide was pilot tested and revised accordingly. KI were initially contacted by email or telephone and invited to participate in the study. All interviews were conducted face-to-face by the first author. All interviews were audio-recorded and transcribed verbatim. The interviewer also took notes during the interviews.

### Analysis

Data were manually analysed following the precepts of content analysis. Key stages in analysis included all authors independently identifying codes from which emergent views were developed and refined. Efforts were made to determine adequacy, credibility, usefulness and consistency of data in relation to the general objective of the study. Where interpretation differed, consensus was achieved through revisiting the raw data and discussions

### Ethical considerations

This study was approved by the Institutional Review Board of the Institute of Tropical Medicine, Antwerp (Belgium) and the Uganda National Council for Science and Technology. All respondents provided informed consent prior to the interviews.

## Results

### Role of evidence

Almost all respondents noted that evidence is important in guiding health policy and programming decisions because it shows what can and cannot work. However, several respondents stated that the use of evidence in policy development was limited and that politics and previous experience played a greater role in policy making. A Ministry of Health (MoH) official noted, *“Currently there is little or no use of evidence, we are relying more on previous experience, politics and previous views on issues. We are developing so many polices that are not evidence based.” A donor similarly remarked that “…policy in Uganda is guided by political influence not research evidence”*.

Some respondents raised a concern of a limited understanding of what evidence is as one of the hindrances to its uptake as elaborated in the following quotes:

*“The challenge is the narrow view of what evidence is. People think that evidence is what has been published in peer reviewed journals and this in a way limits the evidence that goes into our policy process. Evidence could be in a report that was done following a systematic approach”* Donor respondent

*“Evidence is very important for practice but also we need to define what is called evidence. Sometimes evidence to one is not evidence to another. Is it empirical or scientific? We need to know that policy development is sometimes driven by people who even do not sit to formulate the policy so what evidence do you give them?”* CSO respondent.

### Definition of KT

**Figure 1:**
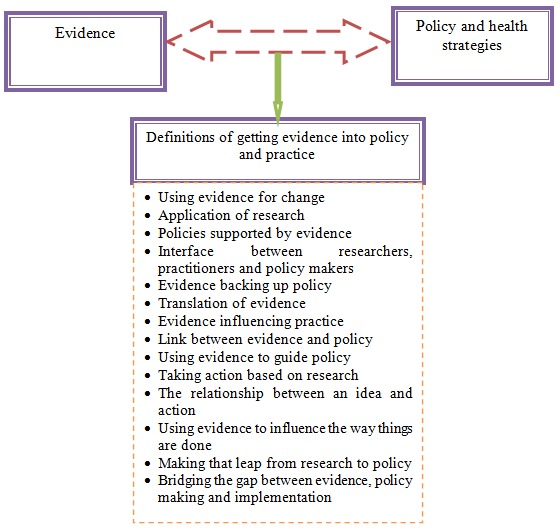
Definitions of getting evidence into policy given by policy actors in Uganda

We noted significant variations in respondents’ conceptualization of KT. The study identified 14 definitions of KT from our respondents (Figure 1). Some respondents defined KT as a relationship between stakeholders, between an idea documented in available evidence and action. Others referred to KT as taking action based on research while others defined it as having policies supported by evidence.

### Involvement in KT activities

Respondents reported having been involved in several KT activities as shown in Table 2. Majority of respondents reported involvement in partnerships at several stages including, research priority setting, undertaking research and policy development where they advocate for the adoption of evidence-based decisions. Partnerships in KT were described by some as difficult to define and implement. A donor respondent remarked that *“What is the best way to involve all stakeholders through the whole process? Should it be through regular updates, should it be in analysis? But how do you ensure independence of researchers?”*

**Table 2:** Number of respondents who reported trying the different KT activities in Uganda

	MoH	Donors	CSO/PNFP)	PFP	Researchers
Building partnerships/participation in partnerships	2	2	4	0	1
Putting platforms in place including researchers, policy makers, CSOs	4	1	2	0	0
Ensuring that Prioritized/commissioned research undertaken/supported	4	1	2		1
Dissemination	0	3	3	1	2
Ensuring MoH leadership in the KT process	1	0	0	0	0
Building capacity of implementers to implement research results	1	1	0	0	0
Demonstration that a given intervention works	0	1	4	0	0
Involving communities in research processes	0	1	0	0	0
Hiring independent credible researchers	0	1	0	0	0
Building basic research skills among stakeholders	0	0	1	0	0

MoH: Ministry of Health; CSO: civil society organization; PNFP: private not for profit; PFP: private-for profit sector

Some respondents reported having put in place platforms for stakeholder engagement; however, the participation in and life span of these platforms varied considerably. Many platforms were implemented as a one off to follow a certain research process through, while one had been in place for a longer duration (up to four years). Platforms for KT were in most cases between two categories of stakeholders for example, civil society and policy makers, civil society and researchers, researchers and policy makers, or donors and policy makers. No respondent reported a platform purposively for KT involving more than two categories of stakeholders. A MoH focal person explained that interactions between researchers and policy makers were often limited to presentations by researchers to an audience of policy makers. Respondents emphasised the need to evaluate the effectiveness of these platforms as illustrated in the following quote by a researcher, *“Previous exercises of bringing policy makers and researchers together have not been reviewed, even WHO has not done any systematic evaluation on effectiveness of KT platforms”*

Undertaking or supporting commissioned research was identified as an activity that facilitated KT because this ensured that research undertaken was addressing information gaps highlighted by policy makers. For example, the MoH had commissioned research on priority areas that had resulted in changes in the logistics systems as highlighted in the quote below from a MoH official:

“Efforts undertaken include commissioning research. We as policy makers’ perceived the need for evidence and commissioned studies. We discussed results in technical fora that bring together researchers and policy makers specifically in the technical working groups (TWG). Here I have several examples where actually research has influenced policy. For example, the study on tracking medicines, we commissioned the research, good quality research was undertaken which was then discussed in the technical working group. This informed development of the medicines logistics system and quantification of medicines requirements”

Dissemination took several modalities including meetings to present research reports, sharing policy briefs with senior health officials, publication in peer-reviewed journals and on websites, and face-to-face discussions with policy makers. Respondents reported that none of these methods worked consistently.

KIs from the CSOs mentioned sharing of evidence from their implementation research where they have demonstrated that a given intervention works. A CSO KI stated that “*In the case of TB/HIV integration, we noted that the programmes were running parallel and thought of ways we could gain from integration. We then decided to pilot integration to see what benefit it has and how it actually works and we got good results. We shared this with policy makers and it was easy to convince them”*

A donor respondent reported successful KT following implementation research stating, “An example is the community HIV/AIDS programme. We demonstrated that it works through implementation research. Although global evidence was available that it was successful, people here were not convinced that it works. We undertook implementation research and we kept addressing problems as they arose, eventually people were convinced that it works.*” Similarly, an MoH official reported, “We piloted injectable depo-provera at the community level, finalised the implementation research process and it is now policy”*

However, in some instances, despite demonstration that a certain intervention works through implementation research, KT has not been successful. For example, a respondent from a CSO stated that although there was evidence demonstrating the feasibility of task shifting to address the human resources for health challenges in Uganda, there has been little success in developing a task shifting policy. Additional efforts to influence the development of a task shifting policy were also unsuccessful as highlighted in the quote below by a researcher: *“We produced policy briefs on task shifting, we went ahead and disseminated them to policy makers and parliamentarians and they were discussed in these fora. This may not have been used as expected at country level (Uganda), but has been taken up by WHO and the global guidelines are being developed on task shifting”*

## Discussion

Our study aim was to increase our understanding of the utilization of evidence in health policy making and programming in Uganda, and to assess stakeholders’ conceptualization of KT and involvement in different KT activities. Majority of respondents agreed that although policies should be informed by evidence, this was not always the case in Uganda.

We noted that respondents had multiple, often limited, definitions of KT. The multiplicity and limited nature of definitions of KT may be a hindrance to KT in Uganda. None of the definitions provided showed an appreciation of understanding of KT as a prolonged process starting with the generation of evidence, synthesis, interpretation and subsequently application. Some respondents defined KT as a link between evidence and policy which infers a notion of a linear model. Previous studies have shown that linear models are not effective [2]. Most definitions were limited to one step in the research generation and application process and in most cases, when results were available [13]. Cordero et al, in their survey of funding agencies supporting KT in low-income settings, also noted a multiplicity of definitions [17].

Although respondents had been involved in several KT activities, the outcomes of these activities varied. Partnerships between stakeholders were frequently mentioned. However, majority of partnerships were short lived and largely involved researchers and policy makers. In contrast to Armstrong et al [6] who emphasised the importance of a two way participation involving translation and exchange amongst stakeholders, we noted that knowledge sharing in these partnerships was mostly uni-directional with researchers sharing their results to an audience of policy makers.

The limited success of partnership may stem from several factors. First, it may imply presence of a very bureaucratic policy making process where the issue of providing evidence and policy development is restricted to a few stakeholders. Indeed, in an earlier study on use of evidence in policy development in Uganda, CSO respondents highlighted the bureaucratic policy making process as a hindrance, citing government restrictions on who participates in certain processes [21]. Second, the limited nature of partnerships could reflect people's understanding of who qualifies to be a stakeholder in KT. For example, a study carried out in Uganda by the COHRED revealed the limited involvement of civil society in health research [22]. Donors, on the other hand, have been shown to have un-due influence [12] and have in some instances required the undertaking of research as a pre-requisite to providing funding [23]. Third, the limited success of partnerships may stem from the challenges of engaging some of the stakeholders. Partnerships are more complex than perceived by the respondents in this study. Successful partnerships take into account varied capacity of stakeholders and the need to invest both time and resources [7]. Bergstrom et al, in their study on the relevance of the Promoting Action on Research Implementation in Health Services (PARIHS) framework in Uganda, identified the importance of community involvement [24] but noted that modalities of engaging the community effectively were not in place [24, 25]. The need to map out all relevant stakeholders and tailored modalities of engaging them has been emphasised in literature [26]. Theobald et al elaborated the need to develop partnerships at multiple levels and with multiple layers within the health system [26].

Respondents identified instances where evidence has informed policy and strategy development, for example, in cases of commissioned research. In the case of demonstration through implementation research, we see a mixed picture. On one hand, evidence demonstrating that a given intervention works may fail to lead to change because of the implementation costs, for example in the case of task shifting [27]. On the other hand, where there is extensive support, successful evidence uptake may occur following implementation research as happened with the community HIV programme. Donor influence may have played a role here, although we did not assess this specifically.

Dissemination of research findings took several forms and was not always systematic and audience-tailored. This could be explained by the nature partnerships reported in this study which may not allow mapping relevant stakeholders and developing tailored messages. The importance of using several modalities for disseminating evidence that are audience-specific has been emphasised [17]. Ineffective dissemination may also stem from the lack of an institutional set up for dissemination of evidence. An earlier study in Uganda highlighted the need to establish a unit within the MoH that would be charged with the responsibility of disseminating evidence [21].

Overall, the effectiveness of KT strategies is highly variable and dependent on the setting. Success hinges on whether the strategies have been sufficiently tailored to target audiences. The effectiveness of the different KT strategies is not known and Cordero et al pointed the need for further research in this area specifically to evaluate KT activities to learn what works, why and in what context [17]. Working through government institutions has been emphasised as a way of ensuring that government takes ownership of the KT process [21]; but, relevant government institutions must be strengthened. In the case of Uganda, the Uganda National Health Research Organization is legally mandated to coordinate KT efforts. However, the institution is not sufficiently resourced to play that role effectively. Inadequate investment in improving KT may partly stem from the low prioritisation of research. Although Uganda recognises the importance of evidence as a critical factor in public health policy development, there is no system in place to track research undertaken and over 90% of health research is funded by external sources whose priorities may not align with country priorities [22]. Use of evidence in public health policy development in resource-constrained settings is of paramount importance because meagre resources must be invested wisely to ensure maximum return. In light of this, investment in KT needs more emphasis.

Study findings should be interpreted in light of the following limitation. The study reported respondents’ perspectives about KT in general and not in reference to on-going specific policies or research project activities. Therefore, responses provided in this study did not refer to a specific research and policy. We note that different KT activities may work for different policies and the generalised responses may not clearly highlight this fact. In this regard, generalised application of our findings may be limited. We however provide a basis for further research on this subject.

## Conclusion

Strategies to improve KT are context-specific. Although a lot of work has been done on KT in high income countries, LIC, where the use of evidence would help countries use limited resources in more effective ways, still face a dearth of context-specific literature on this subject. There is need for conceptual clarity on KT, adoption of a systematic KT framework and, further understanding of the effectiveness of the different KT strategies in low-income settings.
